# Effects of Different Training Interventions on Heart Rate Variability and Cardiovascular Health and Risk Factors in Young and Middle-Aged Adults: A Systematic Review

**DOI:** 10.3389/fphys.2021.657274

**Published:** 2021-04-23

**Authors:** Bernhard Grässler, Beatrice Thielmann, Irina Böckelmann, Anita Hökelmann

**Affiliations:** ^1^Department of Sport Science, Faculty of Humanities, Otto von Guericke University, Magdeburg, Germany; ^2^Department of Occupational Medicine, Faculty of Medicine, Otto von Guericke University, Magdeburg, Germany

**Keywords:** heart rate variability, autonomic nervous system, physical intervention, cardiovascular risk factors, healthy adults

## Abstract

**Introduction:** Heart rate variability (HRV), the beat-to-beat variation of adjacent heartbeats, is an indicator of the function of the autonomic nervous system (ANS). Increased HRV reflects well-functioning of autonomic control mechanism and cardiovascular health. The aim of this systematic review is to provide a systematic overview of the effects of different physical training modalities on resting HRV and cardiovascular health and risk factors (i.e., baroreflex sensitivity, body fat, body mass, body mass index, blood pressure, heart rate recovery, VO_2_ max, and VO_2_ peak) in young and middle-aged (mean age of the studies samples up to 44 years), healthy adults.

**Methods:** A systematic review in accordance with the PRISMA guidelines was performed. Studies investigating the effects of different physical interventions (endurance, resistance, high-intensity, coordinative, or multimodal training) on HRV were included. Trials were considered eligible if the intervention lasted for at least 4 weeks and participants were regarded as general healthy. Five electronic databases were searched from 2005 to September 8th, 2020. The methodological quality of eligible studies was assessed by two study quality and reporting assessment scales (TESTEX and STARD_HRV_). PROSPERO registration number: CRD42020206606.

**Results:** Of 3,991 retrieved records, 26 were considered eligible and analyzed. Twelve studies used an endurance training (of which three included high-intensity sessions), six studies resistance training, four studies coordinative training, two studies high-intensity training, and two studies used a multimodal intervention. Overall, the results showed for all types of intervention an improvement in linear and non-linear HRV parameters and cardiovascular health and risk factors. However, quality assessment revealed some methodological and reporting deficits.

**Conclusion:** This systematic review highlights the benefits of different types of physical training interventions on autonomic function and health parameters in young and middle-aged, healthy adults. In conclusion, higher training intensities and frequencies are more likely to improve HRV. For future studies, we recommend adhering to the criteria of methodological standards of exercise interventions and HRV measurements and encourage the use of non-linear HRV parameters.

## Introduction

Cardiac autonomic control describes the regulation of heart function by the sympathetic and parasympathetic branches of the autonomic nervous system (ANS) (Gordan et al., 2015). Fluctuations in the time intervals between consecutive heart beats are characterized as heart rate variability (HRV) and regarded as an indicator of the functional state of the ANS and the adaptability to internal or external stimuli. These beat-to-beat variations of successive NN intervals are the result of complex interactions between sympathetic and parasympathetic influences on the heart rate (McCraty and Shaffer, 2015; Draghici and Taylor, 2016). Release of norepinephrine triggers sympathetic activation, leading to an increase of heart rate and contractility, an enhancement of conductivity for electrical signals in the heart facilitating physical and mental demands. At rest, however, the parasympathetic activity dominates through release of acetylcholine, slowing down heart rate, reducing its contractility, and inhibiting conduction velocity (Shaffer et al., 2014; Gordan et al., 2015). Parasympathetic activity is important for restorative processes and is the main contributor of fluctuations in heart rate (Thayer et al., 2010). While the parasympathetic activation occurs immediately and has only a short-term effect, sympathetic activation has a time delay of a few seconds, though exerts longer lasting effects (Pumprla et al., 2002; Shaffer et al., 2014). The sympathetic and parasympathetic nervous systems do not work exactly in opposite directions. Instead, they can be active simultaneously, e.g., when parasympathetic activity increases, sympathetic activity does not automatically decrease (Billman, 2013; Shaffer et al., 2014; Ernst, 2017).

Reduced HRV indicates relatively high sympathetic activation, disturbed regulation of the ANS, and inadequate adaptation of the cardiovascular system. It is also a sign of chronic stress, depletion of energy reserves, and autonomic imbalance (Shaffer et al., 2014). Relatively high HRV reflects an adaptive organism, optimal energy reserves, and a good state of autonomic control mechanisms (Thayer et al., 2010; Shaffer et al., 2014; Draghici and Taylor, 2016; Ernst, 2017). Thus, HRV is used as a non-invasive marker to help assessing the risk of sudden death, cardiovascular events, or subsequent mortality in post-infarct patients (Kleiger et al., 1987; Malik et al., 1989; Cripps et al., 1991; Tsuji et al., 1994; DeGiorgio et al., 2010), assessing the degree of diabetic autonomic neuropathy (Voss et al., 2015; Villafaina et al., 2017), as a monitoring tool in athletic training, and to prevent the state of overtraining (Plews et al., 2012; Buchheit et al., 2013; Makivić et al., 2013; Botek et al., 2014).

Regular physical activity reduces risk of cardiovascular diseases and premature mortality, and positively affects cardiovascular health and the functional state of the ANS (Sandercock et al., 2005; Hottenrott et al., 2006; Routledge et al., 2010). Especially aerobic training has been described as an optimal form to reduce risks of cardiovascular diseases and enhancing parasympathetic activity. Numerous studies have shown an increase in HRV after aerobic exercise interventions in healthy adults (Carter et al., 2003; Iwasaki et al., 2003; Tulppo et al., 2003), in elderly (Levy et al., 1998; Schuit et al., 1999; Stein et al., 1999; Albinet et al., 2010), and in people with various diseases (Zoppini et al., 2007; Borghi-Silva et al., 2015; Quiles et al., 2019). However, the effects of other types of physical interventions on HRV is still not clear. Especially, there is no consensus about the effects of combined, multimodal training programs and coordinative training on HRV. We define multimodal training interventions as programs including at least two types of modalities, mainly a combination of aerobic and resistance training. The focus of coordinative training interventions is not the improvement of aerobic capacity but the mediation of emotions and the improvement of sport specific skills. Discrepancies in sample characteristics, training types, and parameters such as duration, frequency, or intensity, and methodological differences in HRV assessment in literature, make it difficult to elucidate the effects on HRV in healthy people. Therefore, a comprehensive overview of the effects of different physical interventions on HRV in healthy adults is required. The objective of the present review is to provide an up-to-date analysis of studies on the effects of different physical interventions on HRV in healthy adults without HRV related diseases in the age of 18–44 years. In fact, regarding the upper age limit of 44 years, the average age of the sample of studies has to be considered. The range of age was chosen to focus on young and early middle-aged adults as they are underrepresented in literature regarding prevention of cardiovascular diseases. Instead, the majority of studies on the effects of exercise training is focused on diseased and/or elderly individuals (Bouaziz et al., 2017; Villafaina et al., 2017; Pearson and Smart, 2018; Raffin et al., 2019; Belvederi Murri et al., 2020; Palma et al., 2020). Investigations with elite athletes were excluded as the primary aim of training programs in professional sports is not to improve HRV but the sport specific performance (Plews et al., 2013). In our review of different training protocols and training types on HRV we distinguish between five types of interventions: endurance (aerobic), resistance, high-intensity, coordinative, and multimodal training programs consisting of at least two types of training modalities. Secondary to HRV, cardiovascular and health risk factors such as baroreflex sensitivity (BR), heart rate recovery after a physical test (HRR), blood pressure (BP), body fat (BF), body mass (BM), body mass index (BMI), and VO_2_ max or VO_2_ peak are considered. Based on literature (Carter et al., 2003; Iwasaki et al., 2003; Tulppo et al., 2003), we hypothesized exercise interventions have a positive influence on HRV and secondary outcomes. In particular, aerobic training should demonstrate a pronounced positive effect on HRV.

## Methods

This systematic review provides a summary and qualitative analysis of recent studies on the effects of various exercise interventions on resting state HRV and selected cardiovascular and health risk factors in young and middle-aged healthy adults. We focused on the descriptive methodological aspects regarding intervention design and HRV recording, processing, as well as analysis. The Preferred Reporting Items for Systematic Reviews and Meta-Analysis (PRISMA) (Moher et al., 2009) was employed to carry out this review. Further, methodological and reporting quality of the studies were analyzed via two quality assessment tools (TESTEX and STARD_HRV_).

### Data Sources and Search Strategy

The electronic databases PubMed, Scopus (Elsevir), SPORTDiscus, Ovid, and Cochrane Library were searched for studies from January 1st, 2005, to September 8th, 2020, using the following terms: (resistance training OR resistance exercise OR strength training OR strength exercise OR aerobic training OR aerobic exercise OR physical training OR physical exercise OR multimodal training OR multimodal exercise OR coordinative training OR coordinative exercise) AND (heart rate variability OR HRV OR cardiac autonomic control OR autonomic function OR parasympathetic activity OR parasympathetic nervous system OR cardiac vagal tone OR autonomic cardiac modulation OR vagus nerve OR vagal tone OR vagal activity).

### Inclusion and Exclusion Criteria

The inclusion criteria for relevant studies were: (1) involving at least 10 healthy subjects aged between 18 and 44 years in average without diseases relevant for HRV analysis in the training group; (2) physical training intervention with a minimum of 4 weeks with eight training sessions; (3) randomized controlled trials, quasi-experimental trials, cross-over controlled trials, or controlled trials without randomization; (4) measurement of at least one HRV parameter at resting position before (pre) and after (post) the intervention through Holter ECG or chest belt; (5) studies with 24-h ECG measurements when a short-term recording segment at resting position was analyzed; (6) full-text in English or German language; and (7) human subjects. Exclusion criteria were: (1) studies with subjects with diagnosis of dementia, mental diseases, neurological diseases, endocrinological diseases (diabetes, thyroid gland disease), cardiac diseases, hypertension, Parkinson, or other health-related diseases; (2) measuring acute exercise effects or HRV during exercise; (3) single-case studies, Review articles, short communications, letters with insufficient information to analyze the results, guidelines, theses, dissertations, qualitative studies, scientific conference abstracts, or studies on animals; (4) 24-h ECG recording without short-term analysis at resting position; (5) HRV assessment through recording the pulse rate manually or through photoplethysmography; and (6) studies with professional athletes.

### Data Collection and Analysis

#### Selection of Studies

Retrieved articles were transferred to the Citavi 6 reference manager (Swiss Academic Software, Wädenswil, Switzerland). Duplicates were removed. Two authors (B.G. and B.T.) independently screened titles and abstracts according to criteria for inclusion and exclusion. Thereafter, the full-text of each relevant article was obtained. The same two authors independently screened the full-text of these articles. If no full-text was available, the authors were contacted. The references of the eligible articles were screened to retrieve further articles. Disagreements were resolved through discussion with a third reviewer (I.B.).

#### Data Extraction

Using the PICOS approach (Liberati et al., 2009) two authors (B.G. and B.T.) independently extracted the following data: sample characteristics (sample size, age, gender), HRV protocol [method (ECG or chest belt), respiration (paced or spontaneous), position (supine, sitting, standing), sampling frequency), extracted HRV parameters, secondary outcomes (BF, BM, BMI, BP, BR, HRR, VO_2_ max, and VO_2_ peak], recording length, characteristics of intervention (type, duration of intervention, sessions per week), and control group.

#### Quality Assessment

The methodological quality of the studies was examined using two assessment tools. The first tool, “Tool for the Assessment of Study Quality and reporting in Exercise (TESTEX) scale,” is designed for exercise training studies (Smart et al., 2015). It assesses study quality and study reporting by using 12 parameters with a maximum score of 15 points. Higher scores represent higher methodological quality. The second tool, “Standard for Reporting Diagnostic Accuracy Studies Guidelines for Heart Rate Variability Research” (STARD_HRV_), is designed to assess the methodological quality of studies with the focus on HRV measurement (Dobbs et al., 2019). This tool includes 25 parameters with a maximum score of 25 points. The two authors (B.G. and B.T.) evaluated the quality of included studies independently. Disagreement was solved by discussion or mediated through a third party (I.B.). Based on our research questions and the included studies, the assessment tools were slightly modified. Tables 1, 2 describes the applied criteria for TESTEX and STARD_HRV_, respectively.

**Table 1:** Description of each modified TESTEX criterion.

	**Evaluation point**	**Points**	**Assessment standard**
1	Eligibility criteria specified	1	Information on inclusion and exclusion criteria. For HRV recording, no medication with cardiac effects
		0.5	Specification of inclusion or exclusion
		0	Not reported
2	Randomization specified	1	Yes
		0	No or not reported
3	Allocation concealment	1	Yes
		0	No or not reported
4	Groups similar at baseline	1	All parameters (gender, age, BMI)
		0.5	Partially
		0	No or not reported
5	Blinding of assessor (for at least one key outcome)	1	Yes
		0	No or not reported
6	Outcome measures assessed in at least 85% of patients	3	Dropout of subjects <15% + report of dropout events + persistent subjects with 85% participation in sessions
		2	2 of the aforementioned
		1	Only persistent subjects with 85% participation in sessions
		0	Nothing or not reported
7	Intention-to-treat analysis	1	Yes
		0	No
8	Between-group statistical comparisons reported	2	Reported for primary (HRV) and secondary endpoint (BMI, BP, VO_2_,)…
		1	Reported for primary endpoint
		0	Not reported
9	Point measures and measures of variability for all reported outcome measures	1	*p*-Values and effect size
		0.5	Only *p*-values
		0	Not reported
10	Activity monitoring in control groups	1	Yes, with protocol
		0	No or not reported
11	Relative exercise intensity remained constant	1	Increase in the process with adaptation to performance (intermediate measurement of the performance parameters)
		0.5	Step-by-step concept planned from the beginning
		0	Constant training during the monitoring period
12	Exercise volume and energy expenditure stated	1	Specification of intensity, frequency, mode and duration of the training with a minimum of: • Endurance training VO_2_ or other equivalent • Resistance training 1RM or other equivalent
		0.5	Specification of intensity, frequency, mode, and duration of the training without VO_2_, 1RM, or other equivalent
		0	Not reported

*BMI, body mass index; BP, blood pressure; HRV, heart rate variability; RM, repetition maximum; VO_2_, oxygen consumption*.

**Table 2:** Description of each modified STARD_HRV_ criterion.

		**Evaluation point**	**Points**	**Assessment standard**
1	Title or Abstract	Identification as a study of validation	1	Reported
			0	Not reported
2	Abstract	Structured summary of study objective, design, methods, results, and conclusions	1	Yes
			0.5	Yes, but not structured
			0	Not reported
3	Introduction	Scientific and practical background, including the intended use of the index device/software	1	Complete, including the application of the HRV method
			0.5	With limitations available
			0	Insufficient background
4		Study objectives and hypotheses described	1	Study objectives and hypothesis
				Study objectives without hypothesis
			0	Not reported
5		Study uses within-subject design	1	Reported
			0	Not reported
6		Intended sample size and how it was determined (e.g., G*Power 3)	1	Reported
			0	Not reported
7		Eligibility criteria including specific restrictions (medical use, gender, age, activity level or BMI)	1	Reported for health, medical use, gender, age, activity, and (BMI)
			0.5	Reported in some criteria <1P
			0	Not reported
8	Methods	Pre-testing guidelines reported (e.g., limitations to caffeine, alcohol, physical activity, etc.)	1	Reported for limitations to caffeine, alcohol and physical activity
			0.5	Reported in some criteria <1P
			0	Not reported
9		Setup of reference standard and index device described in sufficient detail to allow replication (e.g., hardware/software such as brand, electrode configuration, etc.)	1	Sufficient description. A replication is possible
			0.5	Limited description. A replication is partially possible
			0	Insufficient description. Replication is not possible
10		Description of environmental conditions (e.g., temperature, humidity, lights on or off, time of day) and posture	1	Temperature + time of day or same time of day + body position
			0.5	Reported in some criteria <1P
			0	Not reported
11		A stabilization period prior to sampling was described	1	Yes, with information about when and how long
			0.5	Yes, with information about when or how long
			0	Not reported
12		The raw sampling rate and length of collection are described	1	Sampling rate + length of collection
			0.5	Only length of collection
			0	Not reported
13		Acknowledgment of breathing (e.g., controlled or not controlled)	1	Reported
			0	Not reported
14		Description of how estimates or comparison measures were calculated (e.g., ES, LOA, Pearson's *r*, or ICC)	1	Reported
			0	Not reported
15		Reasons for missing data, along with percentage missing (e.g., equipment, persistent ectopy), and how it was handled	1	Reasons for missing data + percentage + handling
			0.5	Reported in some criteria <1P
			0	Not reported
16		Interbeat artifact identification method (e.g., algorithm, manual inspection)	1	Manual inspection of artifacts
			0.5	Automatically without manual correction
			0	Not reported
17		Artifact cleaning methods and percentage of beats corrected	1	Description Method (e.g., smoothing or decimation) and percentage
			0.5	Reported in some criteria <1P
			0	Not reported.
18		Description of metrics used and software/script for HRV calculation (log transformation etc.)	1	Reported
			0	Not reported
19		Specification of frequency bands used and how they were calculated (e.g., Fast Fourier Transform or Autoregressive modeling)	1	Reported
			0	Not reported
20		Baseline demographics of participants	1	Reported
			0	Not reported
21	Results	Mean ± SD along with at least one estimates of precision (e.g., LOA, Pearson's *r*, or ICC)	1	*p*-values and effect size
			0.5	Only *p*-values
			0	Not reported
22	Discussion	Study limitations, (e.g., sources of potential bias, confounding variables, statistical uncertainty, and generalizability)	1	In detail (if necessary also as extra section)
			0.5	Discussed, but not in detail
			0	Not reported
23		Implications for practice, including the intended use	1	Detailed, giving practical recommendations (e.g., clientele, how often), extra section
			0.5	Discussed, but not in detail
			0	No statement or simple statement “We have seen differences and suggest that”
24		Where the full study protocol can be accessed if not fully described	1	Reported
			0	Not reported
25	Other information	Sources of funding and other support; role of funders	1	Information about funding and conflict of interest
			0.5	Funding, conflict of Interest or acknowledgment
			0	Not reported

*BMI, body mass index*.

#### Data Synthesis and Analysis

For each selected study, changes in all HRV parameters and the selected cardiovascular and health risk factors from pre to post intervention were collected to compare for within-subject or between-subject changes, respectively. Increases were marked with an upward arrow, decreases with a downward arrow, and no change with a horizontal arrow. Significant changes, based on the criteria of the studies, were marked with an asterisk.

## Results

### Study Selection

**Figure 1:**
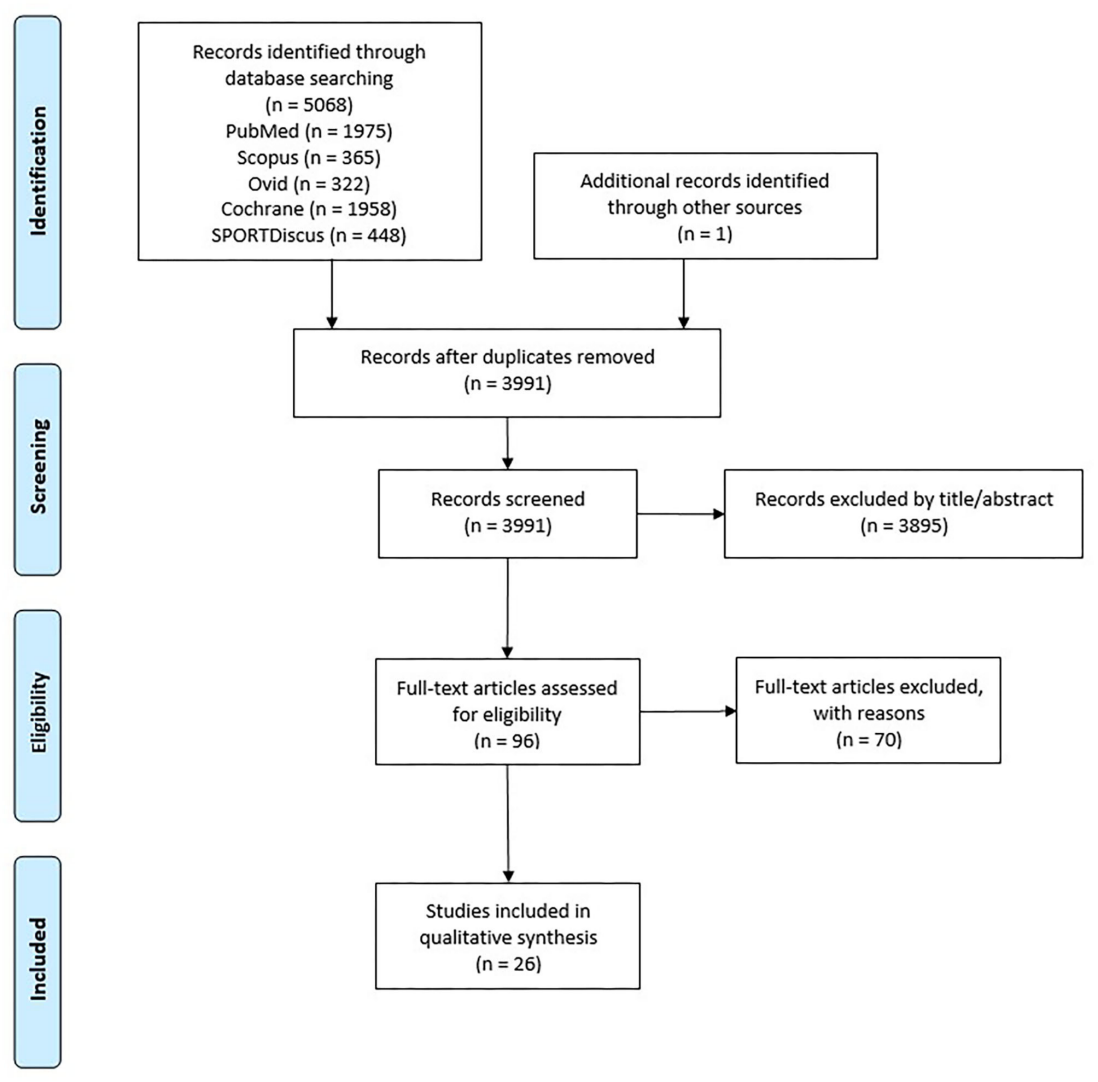
PRISMA flow diagram showing identified, included, and excluded studies.

The initial search identified 5,068 records. After removal duplicates and adding one record through other sources, 3,991 records were screened. Of these, 3,895 were excluded based on title and abstract. The remaining 96 records were assessed for eligibility by full-text assessment of which 26 studies fulfilled the inclusion criteria and thus included in this review. The selection process is shown in the PRISMA flow diagram in Figure 1.

### Study Characteristics

Study characteristics of the eligible studies are summarized in Table 3. A detailed description of variables is available in the electronic Supplementary Table 1.

**Table 3:** Characteristics of the studies included.

**Author, year**	**Participants [sample size, age (year), gender]**	**HRV protocol (method, respiration, position)**	**HR- and HRV-parameters**	**Cardiovascular health and risk factors**	**Recording length**	**Intervention (type, duration, sessions/week)**	**Control group**
Badrov et al., 2013	32 (IHG3: 12; IHG5: 11; CG: 9) Age: IHG3: 23 ± 4; IHG5: 27 ± 6; CG: 24 ± 8 100% female	1-lead ECG Spontaneous Supine 1.000 Hz	RHR, SDNN, RMSSD, pNN50, LF (ln, ms^2^), HF (ln, ms^2^), LF nu, HF nu, TP (ln, ms^2^), LF/HF, SampEn, α1	BP	10 min (256 beats for analysis)	Isometric handgrip training 8 weeks IHG3: 3, IHG5: 5 sessions	Yes
Bond et al., 2009	32 (TG: 16; CG: 16) Age: TG: 20.4 ± 1.9; CG: 22.4 ± 2.2 100% male	2-lead ECG 6 breaths/min n.r. 500 Hz	RHR, HF nu	SBP, VO_2_ peak	5 min	Endurance (bicycle) 8 weeks 3 sessions	Yes
Boutcher et al., 2013	32 (TG: 16; CG: 16) Age: TG: 23.0 ± 4.0; CG: 21.1 ± 2.4 100% female	Polar S810i chest belt Spontaneous Supine n.r.	RHR, LF (ln, ms^2^), HF (ln, ms^2^), LF nu, HF nu	BF, BM, VO_2_ max	15 min	HIIE (cycle ergometer) 12 weeks 3 sessions	yes
Brown et al., 2014	73 (NW: 27; BW: 27, CG: 19) (Baseline: N=94) Baseline: Age: NW: 46.3 ± 9.4; BW: 39.3 ± 10.3; CG: 40.2 ± 11.0 NW: 81% male; BW: 73% male; CG: 83% male	eMotion sensor ECG n.r. Sitting n.r.	RHR, ln HF	BM, BMI, BP, HRR	3 min	Walking 8 weeks 2 sessions	Yes
Buchheit et al., 2010	14 Age: 36 ± 7 100% male	Polar S810 chest belt Spontaneous Supine n.r.	RHR, ln RMSSD (weekly average of 1st and 8th week)	BF, BM, HRR	5 min	Endurance (continuous runs and intervals) 8 weeks 3–4 sessions	No
Cheema et al., 2013	37 (TG: 18; CG: 19) Age: TG: 37 ± 12; CG: 39 ± 13 7 men, 30 women	ECG (Sphygmocor) Spontaneous Supine n.r.	RHR, ln SDNN, ln RMSSD, pNN50, ln HF, ln LF, ln LF/HF, ln TP		10 min	Yoga 10 weeks 3 sessions	yes
Chu et al., 2015	52 (TG: 26, CG: 26) Age: TG: 27.58 ± 5.72; CG: 24.85 ± 5.17 100% female	ECG n.r. Supine 1.024 Hz	LF nu, HF nu, LF/HF		10 min	Yoga 8 weeks 2 sessions	Yes
Cooke and Carter, 2005	22 (TG: 12; CG: 10) Age: TG: 21 ± 0.3; CG: 22 ± 0.7 TG: 1 woman; CG: 3 women	3-lead ECG 15 breaths/min Supine 500 Hz	mRR, SDNN, LF, HF, LF nu, HF nu	BR, SBP	5 min	Resistance (whole body) 8 weeks 3 sessions	Yes (recreationally active)
da Silva et al., 2019	30 (HRVG: 15; CG: 15) Age: HRVG: 25.8 ± 3.1; CG: 27.7 ± 3.6 100% female	Polar RS800CX Spontaneous Standing n.r.	RMSSD	HRR	3 min	Endurance (running) 8 weeks 3 sessions	Yes (predefined training program)
Duarte et al., 2015	40 (TG: 22; CG: 18) Age: TG: TL: 19.2 ± 1.0; TH:19.5 ± 1.2. CG: CL: 19.1 ± 0.3; CH:18.9 ± 0.6 100% male	3-lead ECG Spontaneous Supine 1.000 Hz	RHR, RMSSD, HF nu, LF nu, LF/HF	HRR	5 min	Endurance (running) 12 weeks 3 sessions	Yes
Gamelin et al., 2007	10 Age: 21.7 ± 2.2 100% male	Polar S810 12 breaths/min Supine 1.000 Hz	mRR, SDNN, RMSSD, NN50, pNN50, ln (LF+HF), ln LF, ln HF, LF nu, HF nu, LF/HF, SD1, SD2	VO_2_ max	5 min (last 256 s for analysis)	Endurance (intervals and continuous runs on a treadmill) 12 weeks 2–4 sessions	No
Heffernan et al., 2007	14 Age: 25.0 ± 1.1 100% male	1-lead ECG 12 breaths/min Supine 1.000 Hz	mRR, SDNN, ln TP, ln LF, LF nu, ln HF, HF nu, LF/HF, SampEn, LZEn	BF, BM, HRR, VO_2_ peak	At least 200 NN intervals	Resistance (whole body) 6 weeks 3 sessions	No
Heffernan et al., 2009	39 Age: African Americans: 22 ± 1; Whites: 24 ± 1 100% male	1-lead ECG 12 breaths/min Supine 1.000 Hz	mRR, RMSSD, pNN50, ln TP, ln LF, LF nu, ln HF, HF nu, ln LF/HF, SampEn	BF, BM, BR, HRR, VO_2_ peak	15 min (220–260 s for analysis)	Resistance (whole body) 6 weeks 3 sessions	No
Heydari et al., 2013	38 (TG: 20; CG: 18) Age: 24.9 ± 4.3 100% male	Polar RS800CX Spontaneous Supine 1.000 Hz	RHR, RMSSD, pNN50, ln + ms^2^ (TP HF, LF, VLF)	BM, BMI, BP, BR, VO_2_ max	30 min	HIIE (8 s sprints on cycle ergometer) 12 weeks 3 sessions	Yes
Kim et al., 2017	34 (MAT: 12: HAT: 12; CG: 10) Age: MAT: 24.08 ± 0.62; HAT: 23.25 ± 0.63; CG: 23.30 ± 0.47 100% male	Polar RS800CX 15 breaths/min Sitting 256 Hz	RHR, RMSSD, pNN50, HF, HF nu, LF nu, LF/HF, SD1, SD1/SD2	BMI, BP, VO_2_ max	10 min	Endurance (walking/running on a treadmill) 8 weeks 3 sessions	Yes
Kiviniemi et al., 2010	53 (ST: 14; HRV-I: 14: HRV-II: 10; CG: 15) Age: Men: ST: 37 ± 3; HRV-I: 35 ± 4; CG: 34 ± 4. Women: ST: 34 ± 4; HRV-I: 33 ± 4; HRV-II: 35 ± 4; CG: 34 ± 4 21 men, 32 women	12-lead ECG Spontaneous 2 min sitting and 3 min standing n.r.	RHR, SD1	VO_2_ peak	5 min	Endurance (running, walking or cycling) 8 weeks at least 5 sessions	Yes
Martinmaki et al., 2008	11 Age: 36.8 ± 7.2 100% male	Polar Electro Spontaneous Supine, sitting and standing 1.000 Hz	RHR, TP, HF, LF	VO_2_ peak	3 × 3 min	Endurance (cycling, running, Nordic Walking) 14 weeks 2 sessions	No
McHugh et al., 2012	16 Age: 23.94 ± 5.57 (post) 7 males, 9 females	2-lead ECG 12 breaths/min Supine 1.000 Hz	HF nu, LF/HF	BF, BM, BMI, HRR, VO_2_ max	5 min	Endurance (recreational jogging) 7 weeks 2 sessions	No
Moreira et al., 2017	18 (TG: 10; CG: 8) Age: TG: 25.4 ± 3.3; CG: 29.6 ± 6.3 100% male	Polar RS800CX n.r. Sitting n.r.	RHR, RMSSD, mRR, pNN50, SD1	BP	20 min	Capoeira 10 weeks 1 session	Yes
Nuuttila et al., 2017	24 (HRVG, 13; PD: 11) Age: HRVG: 29 ± 4: PD: 31 ± 5 100% male	Morning: Garmin 920XT; night: Firstbeat Bodyguard Decvice n.r. Supine n.r.	RHR, RMSSD, LF, HF, TP	BF, BM, VO_2_ max	3 min morning, 4 h night	HIT [running and resistance (whole body)] 8 weeks 6 sessions	No
Pal et al., 2014	60 (20-29y, 30–39y, 40–49y; 20 in each group) Age: AG I: 20–29; AG II: 30–39; AG III: 40–50 100% male	ECG (ProComp) n.r. Supine n.r.	RHR, mRR, SDNN, RMSSD, pNN50, HF nu, LF nu, LF/HF, TP	BM, BMI, BP	5 min	Yoga 3 months 6 sessions	No
de Rezende Barbosa et al., 2016	29 (FTG: 13; CG: 16) Age: FTG: 23.0 ± 2.1; CG: 20.6 ± 1.3 100% female	Polar S810i chest belt Spontaneous Supine n.r.	mRR, RRtri, TINN, SD1, SD2, SD1/SD2		20 min	Resistance (whole body) 12 weeks 3 sessions	Yes
Schumann et al., 2017	24 (ST: 14; TLG: 10) Age: 34.0 ± 7.0 100% male	Polar RS800CX n.r. Supine and standing n.r.	RHR, RMSSD, HF, LF, LF/HF (12th vs. 24th week)	BF, BM, VO_2_ max (12th vs. 24th week)	180 s supine, 120 s standing	Endurance (focused on running) 12 weeks 4–6 sessions	No
Sousa Fortes et al., 2018	31 (CLG: 10; MS: 11; CG: 10) Age: CL: 22.04 ± 1.7; MS: 21.72 ± 1.7; CG: 21.85 ± 1.9 100% male	Polar RS800CX n.r. n.r. 1.000 Hz	Ln RMSSD		5 min	Resistance (bench press and leg press) 8 weeks 3 sessions	Yes
Varela-Sanz et al., 2017	31 (TT: 11, PT: 10, CG: 10) Age: TT: 22.36 ± 2.62; PT: 21 ± 2.71; CG: 22.2 ± 2.44 26 men, 5 women	Polar S800 n.r. Supine and standing n.r.	RHR, SDNN, RMSSD, LF, HF	BM, BMI, VO_2_ max (estimated)	n.r.	2x endurance (brisk walking or running) and resistance (bench press and half squat), 1x endurance 8 weeks 3 sessions	Yes
Wu et al., 2011	47 (TG: 22; CG: 25; Follow-up: 17) Age: TG: 21.0 ± 1; CG: 21.0 ± 0.9 100% female	1-lead ECG n.r. Sitting n.r.	RHR, SDNN, RMSSD, LF, HF, LF nu, HF nu	BMI	5 min	Endurance (running on a treadmill) 6 weeks 3 sessions	yes

*AG, age group; BF, body fat; BM, body mass; BMI, body mass index; BP, blood pressure; BR, baroreflex sensitivity; BW, built walk group; CG, control group; CH, control group with high levels of HF at baseline; CL, control group with low levels of HF at baseline; CLG, clustering group; ECG, electrocardiography; FTG, functional training group; HAT, high-intensity aerobic training; HF (nu), power in high frequency range (in normalized units); HIIE, high-intensity intermittent exercise; HRR, heart rate recovery; HRV-I and II, HRV guided training based on daily HRV measurements; HRVG, HRV guided training group; IHG3, isometric handgrip training 3 days per week; IHG5, isometric handgrip training 5 days per week; LF (nu), power in low frequency range (in normalized units); ln, natural logarithm; LZEn, Lempel-Ziv entropy; MAT, moderate-intensity aerobic training; MS, multi-sets group; mRR, mean RR interval; NN50, number of pairs of adjacent NN intervals differing by more than 50 ms in the entire recording; n.r., not reported; NW, nature walk group; PD, predetermined training group; pNN50, NN50 count divided by the total number of all NN intervals; PT, polarized training group; RHR, resting heart rate; RMSSD, square root of the mean of the sum of the squares of differences between adjacent NN intervals; RRtri, HRV triangular index; SampEn, sample entropy; SBP, systolic blood pressure; SD1, standard deviation of instantaneous beat-to-beat variability, extracted from Poincaré Plot; SD2, standard deviation of the long-term variability, extracted from Poincaré Plot; SDNN, standard deviation of NN intervals; ST, standard training group; TG, training group; TH, training group with high levels of HF at baseline; TINN, baseline width of the minimum square difference triangular interpolation of the highest peak of the histogram of all NN intervals; TL, training group with low levels of HF at baseline; TLG, training load-guided (based on estimated cumulated training load); TP, total power; TT, traditional-based group; VLF, power in very low frequency range; VO_2_ max, maximum oxygen consumption; VO_2_ peak, peak oxygen consumption; α1, short-term component of Detrended Fluctuation Analysis*.

The selected 26 studies comprised a total of 843 participants (312 women; 531 men) with 569 in exercising groups and 274 in control groups. Sample sizes ranged from 10 (Gamelin et al., 2007) to 73 subjects (Brown et al., 2014). Eight studies did not include a control group (Gamelin et al., 2007; Heffernan et al., 2007, 2009; Martinmaki et al., 2008; Buchheit et al., 2010; McHugh et al., 2012; Pal et al., 2014; Nuuttila et al., 2017). The mean age of the collated sample was 27.37 ± 7.05 years and ranged between 19.2 (Duarte et al., 2015) and 42 years (Brown et al., 2014). The mean age of the exercising groups was 26.86 ± 6.81 years and ranged from 19.2 (Duarte et al., 2015) to 42.8 years (Brown et al., 2014) whereas the mean age of the control groups was 25.80 ± 6.58 years and ranged from 19 (Duarte et al., 2015) to 40.2 years (Brown et al., 2014). In one trial, only the age range was reported (Pal et al., 2014). Two trials did not differentiate the ages between training and control group (Heydari et al., 2013; Schumann et al., 2017). Six trials included only women (Wu et al., 2011; Badrov et al., 2013; Boutcher et al., 2013; Chu et al., 2015; de Rezende Barbosa et al., 2016; da Silva et al., 2019), 14 trials included only men (Gamelin et al., 2007; Heffernan et al., 2007, 2009; Martinmaki et al., 2008; Bond et al., 2009; Buchheit et al., 2010; Heydari et al., 2013; Pal et al., 2014; Duarte et al., 2015; Kim et al., 2017; Moreira et al., 2017; Nuuttila et al., 2017; Schumann et al., 2017; Sousa Fortes et al., 2018), and six trials included both genders (Cooke and Carter, 2005; Kiviniemi et al., 2010; McHugh et al., 2012; Cheema et al., 2013; Brown et al., 2014; Varela-Sanz et al., 2017). Based on our inclusion criteria, all studies involved healthy participants. Three studies also included a few overweight subjects (Boutcher et al., 2013; Heydari et al., 2013; Brown et al., 2014). In one study, two participants had asthma and one had mild scoliosis (Chu et al., 2015), and another study included only smokers (Kim et al., 2017). Four studies assessed moderately trained (Buchheit et al., 2010), moderately active (Kiviniemi et al., 2010), or recreationally trained subjects (Nuuttila et al., 2017; Schumann et al., 2017).

Half of the trials used an ECG with 1–12 leads and the other half used chest belts to record the NN intervals. The most frequently used recording position was a supine position (19 of 26 studies). Four of these trials also measured ECG at standing position (Kiviniemi et al., 2010; Schumann et al., 2017; Varela-Sanz et al., 2017) and one trial in supine, sitting, and standing position (Martinmaki et al., 2008). In four trials, HRV was recorded in sitting position (Wu et al., 2011; Brown et al., 2014; Kim et al., 2017; Moreira et al., 2017), and in one trial in standing position (da Silva et al., 2019). Two trials did not provide any information about position (Bond et al., 2009; Sousa Fortes et al., 2018).

Spontaneous breathing protocols were used in 10 studies. One study used a paced breathing with six breaths/min (Bond et al., 2009), four studies with twelve breaths/min (Gamelin et al., 2007; Heffernan et al., 2007, 2009; McHugh et al., 2012), and two studies with 15 breaths/min (Cooke and Carter, 2005; Kim et al., 2017). Nine studies did not report their breathing protocol (Wu et al., 2011; Brown et al., 2014; Pal et al., 2014; Chu et al., 2015; Moreira et al., 2017; Nuuttila et al., 2017; Schumann et al., 2017; Varela-Sanz et al., 2017; Sousa Fortes et al., 2018).

Thirteen Studies provided no information about ECG signals sampling frequency. Ten studies (Gamelin et al., 2007; Heffernan et al., 2007, 2009; Martinmaki et al., 2008; McHugh et al., 2012; Badrov et al., 2013; Heydari et al., 2013; Chu et al., 2015; Duarte et al., 2015; Sousa Fortes et al., 2018) recorded ECG with an ideal sampling rate (Sammito and Böckelmann, 2015) of at least 1.000 Hz. One study recorded at 256 Hz (Kim et al., 2017). The recording length was reported in all except one study (Varela-Sanz et al., 2017) and varied between 2 min (Schumann et al., 2017) and 4 h during a nocturnal measurement (Nuuttila et al., 2017). Two studies did not analyze using a fixed time range. Instead, they analyzed at least 200 NN intervals (Heffernan et al., 2007) or an epoch between 220 and 260 s (Heffernan et al., 2009) depending on the number of valid NN intervals. According to recent recommendations, duration of recording should last for at least 5 min to ensure comparability of results across studies and capture the lower frequency waves (Malik, 1996; Laborde et al., 2017).

All studies evaluated cardiac autonomic function using various combinations of time-domain, frequency-domain, or non-linear HRV parameters. Time-domain parameters were assessed in 19 studies, frequency-domain in 20 studies, and non-linear parameters in eight studies. Resting heart rate was assessed in 17 studies, while five studies reported the mean NN interval instead of heart rate (Cooke and Carter, 2005; Gamelin et al., 2007; Heffernan et al., 2007, 2009; de Rezende Barbosa et al., 2016), with two studies reported both (Pal et al., 2014; Moreira et al., 2017). RMSSD or its natural logarithm was the most frequently used time-domain parameter (Gamelin et al., 2007; Heffernan et al., 2009; Buchheit et al., 2010; Badrov et al., 2013; Cheema et al., 2013; Heydari et al., 2013; Duarte et al., 2015; Kim et al., 2017; Moreira et al., 2017; Nuuttila et al., 2017; Schumann et al., 2017; da Silva et al., 2019). HF (ms^2^, nu, or ln) was used in all studies that reported frequency-domain parameters. Regarding non-linear parameters, SampEn (Heffernan et al., 2007, 2009; Badrov et al., 2013), α1 (Badrov et al., 2013), SD1 (Gamelin et al., 2007; Kiviniemi et al., 2010; de Rezende Barbosa et al., 2016; Kim et al., 2017; Moreira et al., 2017), SD2 (Gamelin et al., 2007; de Rezende Barbosa et al., 2016), LZEn (Heffernan et al., 2007), and SD1/SD2 (de Rezende Barbosa et al., 2016; Kim et al., 2017) were reported.

Regarding cardiovascular and health risk factors, we decided to consider only factors, reported in at least three articles: baroreflex sensitivity (BR), blood pressure (BP), body fat (BF), body mass (BM), body mass index (BMI), heart rate recovery (HRR), and VO_2_ max or VO_2_ peak. Four studies did not assess the effects on any health parameter (Cheema et al., 2013; Chu et al., 2015; de Rezende Barbosa et al., 2016; Sousa Fortes et al., 2018). VO_2_ max or VO_2_ peak were the most commonly used factors (13 of 22 studies) (Gamelin et al., 2007; Heffernan et al., 2007, 2009; Martinmaki et al., 2008; Bond et al., 2009; Kiviniemi et al., 2010; McHugh et al., 2012; Boutcher et al., 2013; Heydari et al., 2013; Kim et al., 2017; Nuuttila et al., 2017; Schumann et al., 2017; Varela-Sanz et al., 2017), while only three studies assessed BR (Cooke and Carter, 2005; Heffernan et al., 2009; Heydari et al., 2013).

The majority of trials utilized endurance training interventions (11 of 26 studies) (Gamelin et al., 2007; Martinmaki et al., 2008; Bond et al., 2009; Buchheit et al., 2010; Kiviniemi et al., 2010; Wu et al., 2011; McHugh et al., 2012; Duarte et al., 2015; Kim et al., 2017; Schumann et al., 2017; da Silva et al., 2019). Brown et al. (2014) was also included as endurance training intervention since the effects of a walking program for employees were investigated. Six trials used a form of resistance training (Cooke and Carter, 2005; Heffernan et al., 2007, 2009; Badrov et al., 2013; de Rezende Barbosa et al., 2016; Sousa Fortes et al., 2018), three trials used high intensity training (HIT) (Boutcher et al., 2013; Heydari et al., 2013; Nuuttila et al., 2017), three further trials included HIT sessions (running intervals) in their endurance training programs (Gamelin et al., 2007; Buchheit et al., 2013; da Silva et al., 2019). Interestingly, Kim et al. (2017) used a training group running at moderate (60% of heart rate reserve) and one group running at high intensity (75% of heart rate reserve). Non-standard interventions were also used in a number of studies. These included Yoga (Cheema et al., 2013; Pal et al., 2014; Chu et al., 2015) and Capoeira, a Brazilian martial art/dance (Moreira et al., 2017). Finally, two studies combined endurance and resistance training (Moreira et al., 2017; Nuuttila et al., 2017; Varela-Sanz et al., 2017). Interventions duration ranged from six (Heffernan et al., 2007, 2009; Wu et al., 2011) to 14 weeks (Martinmaki et al., 2008). One (Moreira et al., 2017) to six sessions per week were performed (Pal et al., 2014; Nuuttila et al., 2017; Schumann et al., 2017). A detailed description of the interventions can be found in the Supplementary Table 1.

### Outcome Heart Rate Variability

See Table 4 for the detailed outcome of all HRV measures, cardiovascular and health risk factors, TESTEX, and STARD_HRV_ scores of the included studies. The majority of studies (16/26) reported significant positive adaptations in HRV after physical interventions (Cheema et al., 2013). Five studies showed non-significant improvements (Cooke and Carter, 2005; Martinmaki et al., 2008; Bond et al., 2009; Wu et al., 2011; McHugh et al., 2012; Badrov et al., 2013; Brown et al., 2014; Chu et al., 2015; Schumann et al., 2017). Three studies showed small deteriorations (McHugh et al., 2012; Brown et al., 2014; Chu et al., 2015). Only one study reported significant negative adaptations of HRV after the intervention compared to a control group (Cheema et al., 2013) and one study reported no clear changes in HRV (Schumann et al., 2017). Time-domain parameters improved significantly in 10 of 19 studies reporting them (Buchheit et al., 2010; Heydari et al., 2013; Pal et al., 2014; Duarte et al., 2015; de Rezende Barbosa et al., 2016; Kim et al., 2017; Moreira et al., 2017; Nuuttila et al., 2017; Sousa Fortes et al., 2018; da Silva et al., 2019), frequency-domain parameters in seven of 20 studies reporting them (Gamelin et al., 2007; Boutcher et al., 2013; Heydari et al., 2013; Pal et al., 2014; Duarte et al., 2015; Kim et al., 2017; Nuuttila et al., 2017), and non-linear parameters improved significantly in six of eight studies reporting them (Heffernan et al., 2007, 2009; Kiviniemi et al., 2010; de Rezende Barbosa et al., 2016; Kim et al., 2017; Moreira et al., 2017).

**Table 4:** Outcome of HRV measures, cardiovascular and health risk factors, TESTEX, and STARD_HRV_ score.

**Author, year**	**Between-/Within-group analysis**	**Outcome HR- and HRV-parameters**	**Outcome cardiovascular and health parameters**	**TESTEX**	**STARD _**HRV**_**
Badrov et al., 2013	Within	After 4 weeks: IHG3: ↑ RHR, SDNN, RMSSD, and HF nu; ↓ pNN50, ln LF, LF nu, LF/HF, and α1; ↔ ln HF, SampEn, and TP; IHG5: ↑ SDNN, RMSSD, pNN50, ln LF, ln HF, LF nu, TP, and SampEn; ↓ RHR, HF nu, LF/HF, and α1. After 8 weeks (compared to baseline): IHG3: ↑ SDNN, RMSSD, pNN50, and HF nu; ↓ RHR, ln LF, LF nu, and LF/HF; IHG5; ↔ ln HF, TP, SampEn, and α1; IHG5: ↑ SDNN, RMSSD, ln HF, HF nu, and SampEn; ↓ pNN50, ln LF, LF nu, TP, and LF/HF; ↔ RHR and α1.	Both groups: after 4 weeks: ↓ SBP (IHG3^*^) and DBP; after 8 weeks (compared to baseline): ↓ SBP^*^ and DBP (IHG3); ↔ DBP (IHG5)	12.5	22
Bond et al., 2009	Within	↑ HF nu; ↓ RHR	↑ VO_2_ peak^*^; ↔ SBP	9	19
Boutcher et al., 2013	Within	↓ RHR and LF nu; ↑ HF (ms^2^, nu, ln^*^) and LF (ms^2^, ln)	↑ VO_2_ max^*^; ↓ BF^*^ and BM^*^	6.5	15.5
Brown et al., 2014	Within	↓ ln HF (NW and BW); ↑ RHR (NW and BW)	↑ SBP (BW); ↓ SBP^*^ (NW), DBP^*^ (NW^*^ and BW), BMI, and HRR^*^ (BW and NW)	10	17.5
Buchheit et al., 2010	Within	↑ln RMSSD^*^; ↓ RHR^*^	↓ BF, BM, and HRR	4	17.5
Cheema et al., 2013	Between	↑ ln LF/HF^*^ and RHR; ↓ ln HF, ln SDNN, ln RMSSD, pNN50^*^, ln LF, and ln TP		9.5	18.5
Chu et al., 2015	Within	↑ LF nu and LF/HF; ↓ HF nu		13	21.5
Cooke and Carter, 2005	Within	↑ SDNN, LF, LF nu, HF, and HF nu; ↓ mRR	↑ BR; ↓ SBP (elsewhere published)	9	20
da Silva et al., 2019	Within	Both groups: ↑ RMSSD (HRVG^*^)	Both groups: ↑ HRR (HRVG^*^)	12.5	20
Duarte et al., 2015	Within	↑ RMSSD in TL^*^ and TH, HF nu in TL^*^, LF nu, and LF/HF in TH; ↓ RHR in TL^*^ and TH, HF nu in TH, LF nu, and LF/HF in TL	Both groups: ↑ HRR^*^	11	23
Gamelin et al., 2007	Within	↑ SDNN, RMSSD, NN50, pNN50, ln (LF+HF)^*^, ln LF^*^, ln HF, HF nu, SD1, and SD2; ↓mRR, LF nu, and LF/HF	↑ VO_2_ max^*^	4	18
Heffernan et al., 2007	Within	↑ SDNN, ln LF, LF nu, ln HF, LF/HF, SampEn^*^, and LZEn^*^; ↓ HF nu and mRR; ↔ ln TP	↑ BF, BM, HRR^*^, and VO_2_ peak^*^	4.5	21.5
Heffernan et al., 2009	Within	African Americans: ↑ RMSSD, pNN50, ln TP, ln HF, HF nu, and SampEn^*^; ↓ mRR, ln LF, LF nu, and ln LF/HF. Whites: ↑ RMSSD, pNN50, ln TP, ln LF, LF nu, ln HF, ln LF/HF, and SampEn^*^; ↓ mRR, and HF nu.	African Americans: ↑ BM^*^, BR up-up and down-down, HRR^*^, and VO_2_ peak; ↓ BF. Whites: ↑ BF, BM, BR up-up, HRR^*^, and VO_2_ peak; ↓ BR down-down	4.5	21.5
Heydari et al., 2013	Between	↑ TP, ln TP, VLF, ln VLF, LF, ln LF^*^, ln HF^*^, RMSSD^*^, and pNN50^*^; ↓ RHR^*^, HF, ln HF	↑ BR^*^ and VO_2_ max; ↓ BM^*^, BMI^*^, DBP^*^, and SBP^*^	9	17.5
Kim et al., 2017	Between	MAT: ↑ RMSSD, pNN50, HF, HF nu, SD1 and SD1/SD2; ↓ RHR, LF/HF and LF nu. HAT: ↑ RMSSD^*^, pNN50^*^, HF^*^, HF nu^*^, SD1^*^, and SD1/SD2^*^↓ RHR^*^, LF/HF^*^, and LF nu^*^	MAT and HAT: ↑ VO_2_ max (HAT^*^); ↓ BMI, DBP, and SBP	5.5	16.5
Kiviniemi et al., 2010	Within	↑ SD1 (ST in women, HRV-I in men^*^ and women, HRV-II in women); ↓ RHR (ST in women, HRV-I in men and women, HRV-II in women); ↔ SD1 and RHR (ST men)	All groups: ↑ VO_2_ peak^*^	4.5	15.5
Martinmaki et al., 2008	Within	Supine: ↑ ln HF and ln TP; ↔ RHR and ln LF. Sitting: ↑ ln HF, ln LF, and ln TP; ↓ RHR	↑ VO_2_ peak^*^	6.5	20
McHugh et al., 2012	Within	↑ LF/HF; ↓ HF nu	↑VO_2_ max, HRR (2nd minute after test)^*^; ↓ BF, BM, and BMI	3.5	20.5
Moreira et al., 2017	Within	↑ mRR^*^, RMSSD^*^, pNN50^*^, and SD1^*^; ↓RHR^*^	↓ DBP^*^ and SBP^*^	10	16.5
Nuuttila et al., 2017	Within	Nocturnal: both groups: ↑ RMSSD (HRVG^*^), LF (HRVG^*^), HF, TP (HRVG^*^); ↓ RHR^*^. Morning (estimate from figure): both groups: ↓ RHR^*^; HRVG: ↑ RMSSD; PD: ↔ RMSSD	both groups: ↑ VO_2_ max ^*^. HRVG: ↓BM; ↔ BF. PD: ↔ BM; ↓ BF	10	16.5
Pal et al., 2014	Within	All groups: ↑ HF nu (AG I^*^ and AG III^*^), mRR^*^, RMSSD (AG II^*^ and AG III^*^), pNN50^*^, SDNN^*^, and TP^*^; ↓ LF nu^*^, LF/HF (AG III^*^), and RHR^*^	All groups: ↓ DBP (AG III) and SBP (AG II and AG III^*^); AG I: ↑ BM and BMI; AG II + AG III: ↓ BM (AG III^*^) and BMI^*^	6.5	18
de Rezende Barbosa et al., 2016	Between	↑ RRtri^*^, TINN, SD1^*^, SD2^*^; ↓ mRR^*^ and SD1/SD2		6	19.5
Schumann et al., 2017	Within	ST: supine: ↑ LF; ↓RMSSD, HF and LF/HF; ↔ RHR; stand: ↑ LF and LF/HF; ↓ RHR, RMSSD, and HF (correct value?). TLG: supine: ↑ RMSSD, HF, and LF/HF; ↓ RHR and LF; stand: ↑ RMSSD, HF, and LF/HF; ↓ RHR andLF	ST: ↑ VO_2_ max^*^; ↓ BF^*^ and BM. TLG: ↓ BF and VO_2_ max; ↔ BM	7.5	16
Sousa Fortes et al., 2018	Between and within	CLG and MS: ↑ ln RMSSD^*^ (pre vs. post and vs. CG)		10	16.5
Varela-Sanz et al., 2017	Within	TT: supine: ↑ SDNN, RMSSD, LF, and HF; ↓ RHR^*^; standing: ↑ SDNN, RMSSD and LF; ↓ RHR^*^ and HF. PT: supine: ↑ SDNN and LF; ↓ RHR, RMSSD, and HF; standing: ↑ SDNN, RMSSD, LF, and HF; ↓ RHR	Both groups: ↑ BM (PT^*^), BMI (PT^*^), and VO_2_ max^*^	10.5	16
Wu et al., 2011	Within	↑ RHR, HF, and HF nu; ↓ SDNN, RMSSD, and LF	↓ BMI^*^	6	15

*AG, age group; BF, body fat; BM, body mass; BMI, body mass index; BR, baroreflex sensitivity; BR down-down, BR during hypotensive changes in systolic blood pressure; BR up-up, BR during hypertensive changes in systolic blood pressure; BW, built walk group; CG, control group; CLG, clustering group; DBP, diastolic blood pressure; HAT, high-intensity aerobic training; HF (nu), power in high frequency range (in normalized units); HRR, heart rate recovery; HRV-I and II, HRV guided training based on daily HRV measurements; HRVG, HRV guided training group; IHG3, isometric handgrip training 3 days per week; IHG5, isometric handgrip training 5 days per week; LF (nu), power in low frequency range (in normalized units); ln, natural logarithm; LZEn, Lempel-Ziv entropy; MAT, moderate-intensity aerobic training; MS, multi-sets group; mRR, mean RR interval; NN50, number of pairs of adjacent NN intervals differing by more than 50 ms in the entire recording; NW, nature walk group; PD, predetermined training group; pNN50, NN50 count divided by the total number of all NN intervals; PT, polarized training group; RHR, resting heart rate; RMSSD, square root of the mean of the sum of the squares of differences between adjacent NN intervals; RRtri, HRV triangular index; SampEn, sample entropy; SBP, systolic blood pressure; SD1, standard deviation of instantaneous beat-to-beat variability, extracted from Poincaré Plot; SD2, standard deviation of the long-term variability, extracted from Poincaré Plot; SDNN, standard deviation of NN intervals; ST, standard training group; TH, training group with high levels of HF at baseline; TINN, baseline width of the minimum square difference triangular interpolation of the highest peak of the histogram of all NN intervals; TL, training group with low levels of HF at baseline; TLG, training load-guided (based on estimated cumulated training load); TP, total power; TT, traditional-based group; VLF, power in very low frequency range; VO_2_ max, maximum oxygen consumption; VO_2_ peak, peak oxygen consumption; α1, short-term component of Detrended Fluctuation Analysis*.

* *significant difference (pre vs. post or trainings vs. control group); ↑, increase; ↓, decrease*.

### Outcome Cardiovascular Health and Risk Factors

Significant reductions in blood pressure (systolic and/or diastolic) were detected in five studies (Cooke and Carter, 2005; Buchheit et al., 2010; Badrov et al., 2013; Cheema et al., 2013; Heydari et al., 2013; Brown et al., 2014; Pal et al., 2014; Chu et al., 2015; de Rezende Barbosa et al., 2016; Moreira et al., 2017; Sousa Fortes et al., 2018). VO_2_ max or peak significantly improved in 9 of 13 studies (Gamelin et al., 2007; Heffernan et al., 2007; Martinmaki et al., 2008; Bond et al., 2009; Kiviniemi et al., 2010; Boutcher et al., 2013; Kim et al., 2017; Nuuttila et al., 2017; Schumann et al., 2017). BF, BM, or BMI significantly decreased in five studies (Wu et al., 2011; Boutcher et al., 2013; Heydari et al., 2013; Pal et al., 2014; Schumann et al., 2017). Heart rate recovery was significantly improved in six trials (Heffernan et al., 2007, 2009; McHugh et al., 2012; Brown et al., 2014; Duarte et al., 2015; da Silva et al., 2019). Lastly, BR significantly improved in one study (Heydari et al., 2013). In contrast, BM increased in the investigation of Heffernan et al. (2009). BM and BMI increased in the trial of Varela-Sanz et al. (2017). Four studies did not assess any health parameter of any kind pre and post intervention (Cheema et al., 2013; Chu et al., 2015; de Rezende Barbosa et al., 2016; Sousa Fortes et al., 2018).

### Quality Assessment

The TESTEX scale was used to assess the methodological quality and reporting of the selected studies (Smart et al., 2015). The 26 studies scored from 3.5 (McHugh et al., 2012) to 13 points (Chu et al., 2015) with an average of 7.9 (Table 4). The scoring of each study is detailed in the electronic Supplementary Table 2. However, it has to be mentioned that trials with only one group could not achieve more than eight points. Most studies provided adequate information regarding eligibility criteria, randomization, between-group statistics, and outcome measures. In contrast, most of the studies lacked an intention-to-treat analysis (23/26) and an activity monitoring in the control group (16/21). A subset of studies provided blinding of assessors (Bond et al., 2009; Cheema et al., 2013; Heydari et al., 2013; Chu et al., 2015). Furthermore, only seven of 26 studies achieved three points in subscale number six (“Outcome measures assessed in at least 85% of patients”) (Martinmaki et al., 2008; Badrov et al., 2013; Chu et al., 2015; Nuuttila et al., 2017; Varela-Sanz et al., 2017; Sousa Fortes et al., 2018; da Silva et al., 2019).

The HRV methodology in these studies was assessed with STARD_HRV_ (Dobbs et al., 2019). The scoring of each study is detailed in the electronic Supplementary Table 3. The average score for all studies was 18.44/25 and ranged from 15 (Wu et al., 2011) to 23 points (Duarte et al., 2015). A number of studies failed to provide any information regarding sample size calculation (18/26) and failed to report full information about reasons for missing data, along with percentage missing (24/26), as well as artifact cleaning method and percentage of corrected beats (24/26). Information about pre-testing guidelines, hard- and software they used, stabilization period, and a description of analyzed HRV indices were provided in nearly all studies.

## Discussion

### Purpose and Main Findings

The aim of this systematic review was to summarize the existing literature on the effects of different physical training interventions on HRV in healthy adults. In the current review, we focused on healthy adults aged between 18 and 44 years. The upper age limit concerns the average age of the sample. A variety of physical interventions, such as endurance (aerobic), resistance, high intensity, coordinative, and multimodal interventions were considered. The majority of the studies demonstrated a significant positive effect on HRV after training intervention. Only in one study, the experimental group (Yoga) showed significantly increased LF/HF and reduced pNN50 after the intervention compared to the control group, implying a reduced parasympathetic activity (Cheema et al., 2013). For all five training modalities, significant positive effects on HRV were reported. However, due to the unequal number of studies for each exercise modality, a clear identification of exercise modalities to improve HRV is challenging. Nonetheless, all interventions with HIIT (*n* = 3) and multimodal training (*n* = 2) showed significant improvements in HRV. Four of six studies with resistance training reported significant improvements. Two studies failed to detect any significant change in HRV after the resistance training intervention. Nine of twelve studies with endurance training showed a significant or slightly improved HRV. One study with endurance training did not present clear results and two studies showed a small decline of HRV. Finally, a significant improvement in HRV was shown in two interventions with coordinative training. However, this result was contrasted by two other studies showing an impaired HRV after the same type of modality.

In conclusion, endurance and resistance training are very appropriate exercise modalities to improve resting HRV. Regarding HIIT and multimodal exercise modalities, a definite conclusion cannot be made as supporting data is still limited. For coordinative training, the intensity levels appear to be an important factor in order to stimulate effects on autonomic function. Based on our inclusion of all HRV indices in the respective studies for this review, RMSSD is one of the most used parameters. Further, RMSSD is the most affected parameter after endurance, HIIT, and coordinative training interventions. Therefore, our results are consistent with the model of RMSSD as an indicator of vagally mediated changes in HRV based on the increase of vagal influence after exercise training (Shaffer and Ginsberg, 2017).

Cardiovascular and health risk factors were shown to be improved after the interventions in most of the reviewed studies. Two studies demonstrated an increase of BM (Heffernan et al., 2009) after a resistance intervention and an increase of BM and BMI after a combined endurance and resistance intervention (Varela-Sanz et al., 2017). A number of trials also showed clear deficits in methodological and study reporting quality.

### Endurance Training

#### Heart Rate Variability

Twelve studies applying endurance training interventions were identified. Three of them combined aerobic sessions (continuous runs) with HIT sessions (Gamelin et al., 2007; Buchheit et al., 2010; da Silva et al., 2019). Two studies focused exclusively on women (Wu et al., 2011; da Silva et al., 2019), three studies on women and men (Kiviniemi et al., 2010; McHugh et al., 2012; Brown et al., 2014), and the remaining seven studies included only male participants. While six studies showed significant improvements in at least one HRV parameter (Gamelin et al., 2007; Buchheit et al., 2010; Kiviniemi et al., 2010; Duarte et al., 2015; Kim et al., 2017; da Silva et al., 2019), three trials a tendency of improvement (Martinmaki et al., 2008; Bond et al., 2009; Wu et al., 2011), two trials a tendency of reduced HRV in at least one parameter (McHugh et al., 2012; Brown et al., 2014), one study revealed no clear changes in both training groups from pre to post measurement (Schumann et al., 2017).

The lack of improvement in HRV in two studies could be the result of a relatively low training volume. In the study of Brown et al. (2014), employees had to walk two times per week during their lunch time for about 20 minutes. McHugh et al. (2012) applied a 2-day per week “recreational jogging program”.

Martinmaki et al. (2008) revealed significant improvements in HRV during a submaximal exercise but only very small improvements during rest after the intervention. However, a decrease in resting plasma noradrenaline concentration was found, suggesting a slight decrease of sympathetic control at rest. The unchanged vagal heart rate control could be the result of low training volume (two sessions/week) or the relatively small sample size (*n* = 11). No improvements in HRV at rest but during mental and physical stressors were found in the investigation of Bond et al. (2009) and explained by the lesser vagal withdrawal after the aerobic intervention. Kiviniemi et al. (2010) and da Silva et al. (2019) analyzed the effects of HRV-guided training interventions. The training load in these investigations was determined based on HRV-values. While da Silva et al. (2019) revealed stronger improvements in the HRV-guided group compared to the control group, this effect was only significant in male subjects in the study of Kiviniemi et al. (2010). In contrast, women also could improve their HRV levels. These contrasting results could be explained by the applied sample characteristics. The latter study involved moderately trained subjects with an already good autonomic control, where the former study involved untrained subjects who may have benefitted already from low training loads. Since VO_2_ peak (Kiviniemi et al., 2010) and time to complete running 5 km improved (da Silva et al., 2019) after a HRV-guided training, it is still sensible to monitor autonomic functions by measuring HRV regularly and adapt training load based on the HRV results. In this context, Buchheit et al. (2010) showed an improvement in HRV along with a reduction in time to complete running 10 km. In this study, participants measured their HRV daily. No changes in HRV were detected for the first 4 weeks, showing that 4 weeks are insufficient to improve autonomic function. Hottenrott et al. (2006) recommended at least 3 months of aerobic training to induce modifications in vagal activity. Indeed, 3 months of aerobic training were applied in the studies of Duarte et al. (2015) and Gamelin et al. (2007). Heart rate variability improved significantly in subjects with lower resting vagal activity at baseline but not in subjects with an already high vagal activity (Duarte et al., 2015), confirming the results of Kiviniemi et al. (2010). This implies that autonomic adaptation not only relies on the training volume and intensity but also on the initial autonomic activity of the subject. A significant increase in total power and LF nu in combination with only a moderate increase in HF suggests a quantitative adaptation of autonomic control rather than a qualitative modification of sympathovagal balance (Gamelin et al., 2007). However, the small sample size (*n* = 10) is one limitation of this trial.

In summary, endurance training is appropriate to increase HRV. Higher intensities (Kiviniemi et al., 2010; Kim et al., 2017) and a combination of moderate- and high-intensity interventions (Gamelin et al., 2007; Buchheit et al., 2010; da Silva et al., 2019) seems to be appropriate to improve autonomic function. Since these interventions mainly applied running or running on a treadmill, we recommend running as an appropriate type of intervention. In contrast, low-intensity or low-volume training is not sufficient to significantly improve autonomic function (Martinmaki et al., 2008; Bond et al., 2009; Wu et al., 2011; McHugh et al., 2012; Brown et al., 2014). Compared to lower intensities, high-intensity sessions stimulate nitric oxide syntheses stronger, resulting in a stronger vagal activity (de Abreu et al., 2019). Furthermore, exercise training suppresses angiotensin II expression and thus improves cardiac vagal activity (Routledge et al., 2010). Consequently, to achieve improvements in autonomic functioning, the performance level, HRV at baseline, and HRV behavior in response to training have to be considered (Hottenrott et al., 2006; Kiviniemi et al., 2006). The role of gender in the improvement of autonomic functions needs to be further investigated, as only two studies exclusively examined female subjects. The majority of studies with male participants showed an improvement in HRV. Similarly, the study of Kiviniemi et al. (2010) showed a significant improvement in male participants but only a slight improvement in female participants. This finding could also be due to the already higher vagal activity in premenopausal women compared to men of the same age (Kuo et al., 1999).

#### Cardiovascular Health and Risk Factors

Cardiovascular and health risk factors could be significantly improved upon in all studies with endurance training, except in Buchheit et al. (2010). Here, BF, BM, and HRR showed only a slightly decrease. However, since moderately trained runners were participants, no effects regarding BF and BM were expected. Heart rate recovery was significantly improved in all of the four studies investigating this factor (McHugh et al., 2012; Brown et al., 2014; Duarte et al., 2015; da Silva et al., 2019). A rapid decline in heart rate after a (sub)maximal exercise could be the result of an improved vagal reactivation after an endurance intervention. Duarte et al. (2015) recorded RMSSD after a maximal treadmill test and found significant improvements, indicating that indices of vagal reactivity after exercise would be more sensible for autonomic adaptations after physical interventions than resting HRV alone.

Blood pressure was significantly lowered only in Brown et al. (2014). In addition, BF (Schumann et al., 2017) and BMI (Wu et al., 2011) were significantly reduced only in these studies whereas the other studies failed to report any significant changes. This could be due to the healthy sample pool, as in this review only relatively young subjects without severe health problems were considered. Therefore, a reduction of these risk factors, was not expected.

In contrast, VO_2_ max or peak were shown to be improved in six studies (Gamelin et al., 2007; Martinmaki et al., 2008; Bond et al., 2009; Kiviniemi et al., 2010; Kim et al., 2017; Schumann et al., 2017). McHugh et al. (2012) failed to show significant improvements possibly due to the low training volume (7 weeks, two sessions/week) and low intensity (recreational jogging).

In summary, a few health parameters could be improved upon in nearly all studies, though healthy and relatively young subjects (<45 years) participated in these studies. These improvements were accompanied by improvements in autonomic function and enhanced running performance (Gamelin et al., 2007; Schumann et al., 2017; da Silva et al., 2019).

### Resistance Training

#### Heart Rate Variability

Six studies applying resistance training are included in this review. Two studies included female participants only (Badrov et al., 2013; de Rezende Barbosa et al., 2016), one study included both genders (Cooke and Carter, 2005), and the remaining three studies examined only male subjects (Heffernan et al., 2007, 2009; Sousa Fortes et al., 2018). While two studies reported no significant improvements in HRV (Cooke and Carter, 2005; Badrov et al., 2013), four studies revealed significant improvements (Heffernan et al., 2007, 2009; de Rezende Barbosa et al., 2016; Sousa Fortes et al., 2018).

Isometric handgrip training (IHG) failed to induce HRV modifications, possibly due to its localized effect on handgrip strength (Badrov et al., 2013). Heart rate variability was also unaffected after strength training intervention in the study of Cooke and Carter (2005). Critically, aerobic fitness was not assessed at baseline. Some subjects may have been aerobically fit and therefore differed in their baseline autonomic response. This, in combination to the small sample size of the training group (*n* = 12) may have caused to show a significant improvement on HRV.

Significant improvements in HRV were observed in non-linear parameters but not in time- or frequency-domain indices in the studies of Heffernan et al. (2007, 2009). These findings support the hypothesis of Bhati et al. (2019), that non-linear measures are more sensitive for detecting training-induced autonomic adaptations. The authors supposed an increase of autonomical mediated heart rate complexity (i.e., an increase of the unpredictability of the heart rate time series) after resistance training, reflecting increased parasympathetic and/or reduced sympathetic cardiac autonomic control (Heffernan et al., 2007). de Rezende Barbosa et al. (2016) reported significant improvements in geometric indices following a whole-body resistance training in young women compared to a control group. Sousa Fortes et al. (2018) showed a significant increase of ln RMSSD and a decrease of resting heart rate after two different methods in resistance training (clustering vs. multi-sets) during a 8-week intervention with three sessions per week.

While all three studies with male subjects found improvements in HRV (Heffernan et al., 2007, 2009; Sousa Fortes et al., 2018), the studies with both female and male subjects (Cooke and Carter, 2005) and with only female subjects showed no clear results (Badrov et al., 2013). These findings may indicate that women respond to resistance training with a less pronounced improvement in autonomic functioning compared to men. However, as the studies with endurance training already showed, the higher vagal activity of female subjects before menopause could also be a reason for a lack of training-induced improvement in HRV. Again, more extensive studies with participants comprised of young men and premenopausal women are necessary to investigate gender-specific effects of physical activity on HRV.

Adaptations in autonomic function can be caused by several possible physiological mechanism. For example, reduction in concentration of metabolites and pro-inflammatory cytokines is one mechanism (Sousa Fortes et al., 2018). The optimization of parasympathetic activation and reduction of sympathetic tone, explained by a decrease in plasma concentration of norepinephrine, is another explanation for the increase of HRV following resistance training (Sousa Fortes et al., 2018). Enhanced responsiveness of the baroreflex, vascular adjustments, and increased nitric oxide bioavailability may also cause positive adaptations (Selig et al., 2004; Macedo et al., 2016). Moreover, changes in body composition and enhanced lactate tolerance can also positively influence autonomic function (Bhati et al., 2019).

#### Cardiovascular Health and Risk Factors

Systolic blood pressure was significantly reduced following IHG, regardless of the number of sessions per week (Badrov et al., 2013). In contrast to earlier studies, no reductions in diastolic and mean arterial blood pressure were found. However, Badrov et al. (2013) argued that in these studies the subjects had higher initial BP-values. Isometric handgrip training may be a reasonable method in the prevention of hypertension. Heart rate recovery was improved in two studies and related to a prompt reactivation of parasympathetic activity and sympathetic withdrawal after the exercise (Heffernan et al., 2007, 2009). The authors suggested an increased acetylcholine release from the vagus, increased number of receptors, and their improved sensitivity, induced by resistance training, as possible mediating mechanisms. The small increase of VO_2_ peak was regarded as not physiologically relevant, although a modest improvement of aerobic capacity was possible (Heffernan et al., 2007). A significant increase in BM as reported in Heffernan et al. (2009) could be the result of a hypertrophic effect of the resistance training without detrimental health risks.

### High Intensity (Interval) Training

#### Heart Rate Variability

HIIT on a cycle ergometer was used in two studies (Boutcher et al., 2013; Heydari et al., 2013). Both showed positive effects on vagally mediated HRV. The suggestion of that higher intensities cause an increased effect on HRV has been confirmed by other endurance interventions that included running HIT sessions (Gamelin et al., 2007; Buchheit et al., 2010; Kim et al., 2017; da Silva et al., 2019). Significant improvements were detected in short-term recordings (Gamelin et al., 2007; da Silva et al., 2019) as well as in longer recordings (Heydari et al., 2013). However, none of these studies used ECG, only chest belts for recording the NN intervals. As already mentioned previously, compared to continuous training, high intensities produce a stronger stimulus on nitric oxide syntheses, thus favoring the bioavailability of it and induce greater distensibility of the carotid artery, favoring baroreflex sensitivity (de Abreu et al., 2019). The results here also demonstrate that both women (Boutcher et al., 2013) and men (Heydari et al., 2013) can benefit from high-intensity training. However, interventions with participants of both genders would more clearly demonstrate a gender-specific adaptations to HIIT.

#### Cardiovascular Health and Risk Factors

VO_2_ max was significantly improved upon in two trials (Gamelin et al., 2007; Boutcher et al., 2013). It is supposed that HIIT increases stroke volume by enhanced cardiac contractility, enhanced mitochondrial oxidative capacity, and increased skeletal muscle diffuse capacity (Wisløff et al., 2007; Boutcher et al., 2013; Boutcher and Boutcher, 2017). HIIT is also suitable to reduce BF and BM in overweight women (Boutcher et al., 2013), as well as to reduce BP and improve BR in inactive young men (Heydari et al., 2013). These positive adaptations could be the result of an enhanced vasodilatory capacity, endothelial adaptation, or increase in endothelial NO synthase activity (Heydari et al., 2013).

### Coordinative Training

#### Heart Rate Variability

Regarding coordinative training interventions, three studies applied Yoga (Cheema et al., 2013; Pal et al., 2014; Chu et al., 2015) and one study Capoeira (Moreira et al., 2017). While one study failed to detect any significant changes in female participants (Chu et al., 2015), HRV of male participants improved in one study (Pal et al., 2014), and decreased significantly in another study comprised of men and women (Cheema et al., 2013). In the last study, ln LF/HF increased and pNN50 decreased following 10 weeks of Yoga. The authors explained this result with a short intervention, small sample size, and low statistical power. Although the duration of intervention was shorter and the number of sessions per week even lower, no significant decline but a stable HRV was detected in Chu et al. (2015). These contradictory results are in line with a meta-analysis, showing no strong evidence for the effectiveness of yoga to improve HRV (Posadzki et al., 2015). A longer intervention and higher volume with six sessions per week might be appropriate to induce positive adaptations of the ANS (Pal et al., 2014). The authors concluded that yogic practice improves autonomic function coinciding with increased levels of serotonin and dopamine, and lowered levels of epinephrine and norepinephrine.

One session of Capoeira per week for 10 weeks was enough to increase HRV in young men (Moreira et al., 2017). These improvements may be caused by the additional benefits such as lowering psychological stress and anxiety, socialization, friendship, and relaxation promoted by the capoeira rhythm (Moreira et al., 2017). The inconsistent results could also be due to the gender of the subjects, as in both studies with positive effects on HRV, only men participated, whereas in the intervention of Chu et al. (2015) only women were examined or the majority of participants were women in Cheema et al. (2013).

To summarize, the HRV benefits from coordinative training may be the result of psychological factors, such as social contacts and the elicitation of positive emotions. These factors should be further investigated in future studies with interventions of subjects suffering from psychological problems, and programs with adequate volume and intensity, challenging the cardiovascular system. Additionally, cardiovascular effects could be elicited through the sum of acyclic movements in the coordinative programs. These aspects should be carefully considered, when planning physical interventions with untrained subjects.

#### Cardiovascular Health and Risk Factors

The reduction of BP after the Capoeira intervention may be due to increased parasympathetic tone and reduced sympathetic nerve activity. This is of relevance, since participants showed pre-hypertension prior to the intervention (Moreira et al., 2017). Improvement of BP was also detected after the Yoga intervention in the study of Pal et al. (2014) and was accompanied by improvements of other risk factors (levels of catecholamine, BDNF, and cholesterol).

### Multimodal Training

#### Heart Rate Variability

Nuuttila et al. (2017) investigated the effects of an HIT consisting of endurance training with low- and high-intensities and strength training sessions in young men. Strength training was comprised of maximal and explosive exercises. Therefore, we considered this intervention as a multimodal training. In addition, the authors compared two training groups: HRVG (HRV-guided) and PD (predetermined training group). Heart rate variability was recorded at night (a 4 h block was analyzed) and every morning. The morning measurement was averaged weekly. The results showed significant improvements of the nocturnal HRV in the HRVG. The improvements in the PD were not significant. RMSSD of the morning measurements improved in HRVG and was stable in PD. These results support earlier findings of da Silva et al. (2019). In contrast, Varela-Sanz et al. (2017) could detect no significant improvements in HRV, neither during standing nor supine position in a pool of 30 men and five women. A decline in resting heart rate was the only significant result of this study. The authors argued, that training frequency and duration may have been too low to provoke changes in HRV. In addition, participants were already physically active before the intervention. Thus, higher intensities would be necessary to induce a vagal enhancement.

#### Cardiovascular Health and Risk Factors

VO_2_ max was improved following the HIT in both groups of the intervention in Nuuttila et al. (2017). This enhancement was accompanied by an improved performance in a 3,000 m running test. Therefore, HRV-guided HIT is an effective method to improve autonomic function and running performance. BM and BMI increased after the multimodal training program in the study of Varela-Sanz et al. (2017) but BF remained unchanged. This resulted in an overall improved maximum strength (Varela-Sanz et al., 2017).

### Quality Assessment

#### TESTEX

The methodological quality of the studies ranged from 3.5 to 13, with an average score of 7.90. When trials without a between-group analysis (six studies) were excluded, the score ranged from 4.5 to 13, with an average score of 8.93. The following criteria were fulfilled by most of the studies: specification of the eligibility criteria and randomization, similar groups at baseline, and providing point estimates for all outcomes. However, allocation concealment, blinding of assessors, outcome measures (including reporting adverse events and percentage of exercises completed), intention-to-treat analysis, and activity monitoring in the control groups were fulfilled by only a small number of studies. Therefore, future studies should adhere to these methodological recommendations more closely to allow for better cross-comparison between studies.

#### STARD_HRV_

STARD_HRV_ was devised as an assessment tool for trials measuring HRV. The score of the included studies ranged from 15 to 23, with an average score of 18.44. The first three criteria (identification as a study of validation; structured summary; and scientific and practical background) are fulfilled in all studies. In addition, a description of how estimates were calculated and baseline demographics of participants were provided in all studies. Main deficits were detected in the description of environmental conditions and in the ECG processing (reasons for missing data, along with percentage missing and how it was handled; artifact identification method; artifact cleaning method and percentage of beats corrected). For future studies, we recommend to follow the criteria as stated in TESTEX and STARD_HRV_ to overcome methodological limitations. This will allow better comparability between studies and a standardization of HRV measurement.

### Strengths and Limitations

Several limitations to this review have to be mentioned. First, not only randomized controlled trials were included to provide a broad overview of existing literature. Though, we want to emphasize that randomization is an extremely important aspect in clinical trials. Second, a meta-analysis could provide a quantitative analysis of studies and comparison of the effects on HRV between different types of interventions. However, a quantitative analysis is limited by the heterogeneity of studies, in relation to the exercise intervention protocols, and methods of HRV analysis. Therefore, we decided to refrain from a meta-analysis and conducted a qualitative analysis instead. The applied methodological assessment tools identified some deficits as none of the studies scored the maximum points for TESTEX or STARD_HRV_. This highlights the urgent need of complying to already existing criteria for HRV measurements and exercise training intervention studies. Third, the study sample pool was restricted to healthy adults up to the age of 44 years in average. The conclusions presented here are not generalizable to older adults or diseased samples. Fourth, although we did not limit our literature search to a single gender, only six studies were found that examined exclusively female subjects and six studies that examined female and male subjects together. Additionally, the gender distribution was not balanced in all of these studies. Lastly, only original articles in English and German (no article in German was included in the final sample) were included.

To our knowledge, this is the first systematic review dedicated to examining the effects of a broad range of physical interventions on autonomic function and selected cardiovascular and health risk factors in healthy adults. A key aspect of our review is the inclusion of different training modalities. Although, prevention of cardiovascular and health risk factors is typical in older age ranges, most of the included studies showed already an improvement of autonomic function and cardiovascular and health risk factors in relatively young and untrained adults. Our results highlight the potential of different types of physical modalities to improve autonomic function and to prevent cardiovascular diseases. As measuring HRV at rest alone is not sufficient to evaluate autonomic adaptation, we also considered investigations measuring cardiovascular and health risk factors providing a more comprehensive view of the ANS and cardiovascular system (Bhati et al., 2019).

### Practical Implications

This review highlights the potential of different physical training interventions to improve HRV and health parameters (e.g., reducing blood pressure or increasing VO_2_ max) in young and middle-aged adults. Although aerobic training is the most common modality to improve autonomic functions (Routledge et al., 2010), resistance and high-intensity training interventions improve autonomic function as well (Bhati et al., 2019; de Abreu et al., 2019). Even coordinative training, with adequate exercise frequency and intensity, can enhance HRV (Pal et al., 2014; Moreira et al., 2017). A recent meta-regression has proved training frequency as a key factor influencing HRV positively in older adults. Due to the higher cardiovascular risk induced by higher intensities in the elderly, interventions with elderly should focus on training frequency, rather than intensity (de Abreu et al., 2019; Raffin et al., 2019). However, based on our results, we would recommend higher intensities for healthy, young people. The huge variety in methodological quality highlights the necessity to adhere to quality standards as a critical factor to minimize possible confounding factors and increase the comparability between studies (da Silva et al., 2014).

Since we were not able to formulate an optimal dose of exercise training for enhancing HRV, future studies should investigate the effects of different training doses. Currently, resting and post-exercise HRV are used to monitor the training progress (Makivić et al., 2013) and in the assessment of overreaching and to prevent overtraining (da Silva et al., 2015). Future studies could also employ HRV as an indicator of internal load to directly prescribe exercise intensity (Herold et al., 2020). Non-linear indices, such as DFA1, appear to be potential markers for diagnostics and monitoring of the training process (Gronwald et al., 2020). Therefore, we recommend to utilize not only traditional linear HRV parameters but also to analyze non-linear parameters to gain further insight into the complexity of the ANS. Due to the predominance of studies with male participants, the positive effects of any physical activity on HRV in men can be confirmed. The influence of physical activity in women needs further investigation since studies with exclusively female subjects and studies comparing male and female subjects are the minority. Therefore, this gender gap has to be closed and we encourage to investigate the influence of endurance, resistance, coordinative, multimodal, and HIT in premenopausal women. The effects of the hormonal balance and the menstrual cycle on autonomic modulation should also be considered.

## Conclusion

As hypothesized, this review demonstrated the positive effectiveness of different physical training modalities on autonomic function in young and early middle-aged healthy adults, as indicated by an increase of different HRV parameters at rest. In addition to an increased HRV, selected cardiovascular, and health risk factors (baroreflex sensitivity, body fat, body mass, body mass index, blood pressure, heart rate recovery, and VO_2_ max and peak) improved as well. Several physiological mechanisms are involved in the adaptation of autonomic function. Aerobic training is supposed to decrease plasma noradrenaline concentration resulting in a decrease of sympathetic influence on heart rate (Kiviniemi et al., 2010; Kim et al., 2017). Furthermore, suppression of angiotensin II expression leads to an increase of the parasympathetic influence on the heart rate (Routledge et al., 2010). Additional high-intensity sessions favor nitric oxide synthesis and induce greater distensibility of the carotid artery, which improves baroreflex functional capacity (Bhati et al., 2019). Finally, the reduction of sympathetic tone by decreased plasma concentration of norepinephrine as well as the reduced concentration of metabolites (such as ammonia and urea) and pro-inflammatory cyokines (such as TNF-alpha and interleukin 2) are suggested as probable mechanisms for HRV improvement after resistance training. However, the underlying mechanisms have to be elucidated in future studies. Although we did not assess the quantitative effect of training intensity and frequency on HRV and health parameters by conducting a meta-analysis, our observations strongly suggested higher training intensities and frequencies to improve HRV. However, we recommend for future studies to adhere to the criteria of methodological standards for exercise training studies and HRV measurements and encourage to investigate the gender-related differences in the adaptation of autonomic function.

## Data Availability Statement

The original contributions presented in the study are included in the article/Supplementary Material, further inquiries can be directed to the corresponding author/s.

## Author Contributions

BG developed the concept for this review and designed the review process. BG and BT performed the literature search and analysis. BG wrote and edited the manuscript. BT, IB, and AH reviewed the drafted versions. All authors contributed to the article and approved the submitted version.

## Conflict of Interest

The authors declare that the research was conducted in the absence of any commercial or financial relationships that could be construed as a potential conflict of interest.
